# Comprehensive Evaluation of Probiotic Property, Hypoglycemic Ability and Antioxidant Activity of Lactic Acid Bacteria

**DOI:** 10.3390/foods11091363

**Published:** 2022-05-08

**Authors:** Hongyu Wang, Liang Li

**Affiliations:** 1 State Key Laboratory of Dairy Biotechnology, Shanghai Engineering Research Center of Dairy Biotechnology, Dairy Research Institute, Bright Dairy & Food Co., Ltd., Shanghai 200436, China; bx53314218@163.com; 2 College of Food Science, Northeast Agricultural University, Harbin 150030, China

**Keywords:** probiotic property, hypoglycemic ability, antioxidant activity, lactic acid bacteria, comprehensive evaluation

## Abstract

Taking lactic acid bacteria is an important strategy to alleviate or prevent diabetes, but the candidate strains with good genetic stability and excellent functions still need to be supplemented. In this study, the hypoglycemic ability (α-amylase, α-glucosidase and dipeptidyl peptidase 4), probiotic property and antioxidant activity of lactic acid bacteria were comprehensively evaluated by a principal component analysis (PCA) and analytic hierarchy process (AHP). The results showed that *Lactobacillus paracasei* *(L. paracasei*) had a higher survival rate (82.78%) in gastric juice and good tolerance to bile salt, and can be colonized in HT-29 cells. *L. paracasei* had a remarkable inhibitive activity of α-amylase (82.21%), α-glucosidase (84.29%) and dipeptidyl peptidase 4 (42.51%). *L. paracasei* had better scavenging activity of free radicals, total antioxidant activity (FRAP) and superoxide dismutase activity. According to the scores of the PCA, *L. paracasei* had the best hypoglycemic ability, and *Lactococcus lactis* (*L. lactis*) had the highest probiotic property. According to AHP, *L. paracasei* was the best potential hypoglycemic probiotic; furthermore, *L. lactis* showed the highest comprehensive performance except *Lactobacillus*. All lactic acid bacteria in this test had good safety. *L. paracasei* is expected to become a new potential hypoglycemic strain.

## 1. Introduction

Diabetes is one of the most common chronic diseases due to inadequate (or relative) systemic endocrine insulin, which is predicted to rise to 642 million by 2040 [1]. Type 2 diabetes (T2D), also known as “non insulin dependent diabetes”, accounts for about 90–95% of all diabetes [2]. Patients suffer from persistent hyperglycemia and diabetes-related complications, such as diabetic foot, retinopathy, nephropathy, neuropathy and macrovascular diseases [3,4,5]. Drug therapy is the main way to deal with diabetes at present, which may lead to complications such as hyperglycemia and cardiovascular disease and side effects such as flatulence, abdominal pain and diarrhea [6,7,8]. So, there is an urgent need for a more healthy and effective method.

Lactic acid bacteria (LAB) fall into the category of generally regarded as safe (GRAS) bacteria, and are a kind of commonly used probiotic [9]. Studies have shown that LAB can alleviate diabetes by regulating intestinal microbiota, reducing intestinal leakage, reducing insulin resistance, alleviating oxidative stress, improving insulin secretion and protecting β cells [10,11,12,13]. Many LAB strains have been proven to have potential hypoglycemic abilities in vitro, such as the inhibition of α-amylase, α-glucosidase and dipeptidyl peptidase 4 [14,15,16,17]. It is worth noting that reactive oxygen species (ROS) were proven to be related to disorders of glucose metabolism [18], and oxidative stress is considered to be an important factor leading to T2D [19]. Therefore, the antioxidant activity of lactic acid bacteria is one of the important abilities for alleviating diabetes. According to previous studies [14,15,16,17], LAB had great potential in the treatment of T2D, but the strains with good genetic stability and excellent functions are still very limited; therefore, it is necessary to screen more optional hypoglycemic probiotics.

The purpose of this study was to screen potential hypoglycemic LAB by comprehensively analyzing the probiotic properties, hypoglycemic ability and antioxidant activity of LAB. The evaluation mainly included the inhibition of related enzyme activities (α-amylase, α-glucosidase and dipeptidyl peptidase 4), antioxidant activity and probiotic properties. The analytic hierarchy process (AHP) and principal component analysis (PCA) were used to comprehensively evaluate the properties of hypoglycemic probiotics.

## 2. Materials and Methods

### 2.1. Strains

The strains used in this study were *Lactobacillus plantarum* (*L. plantarum*), *Lactobacillus rhamnosus* (*L. rhamnosus*), *Lactobacillus acidophilus* (*L. acidophilus*), *Lactobacillus delbrueckii* (*L. delbrueckii*), *Lactobacillus paracasei* (*L. paracasei*), *Lactobacillus casei* (*L. casei*), *Streptococcus thermophilus* (*S. thermophilus*), *Leuconostoc mesenteroides* (*L. mesenteroides*) and *Lactococcus lactis* (*L. lactis*). The strains were isolated from traditional Chinese dairy foods, and identified by high-throughput sequencing and stored in the Food Science College of Northeast Agricultural University.

### 2.2. Materials

Pepsin (1:10,000), oxgall, porcine α-amylase and acarbose were purchased from Shanghai yuanye Bio-Technology Co., Ltd. (Shanghai, China). Sodium thioglycolate (THIO) and 2,2-Diphenyl-1-picrylhydrazyl (DPPH) were purchased from Shanghai Macklin Biochemical Co., Ltd. (Shanghai, China). Dulbecco’s modified eagle medium (DMEM) with high-glucose, fetal bovine serum and Penicillin-Streptomycin were purchased from Hyclone (Thermo, Beijing, China). α-Glucosidase, pNPG and DPP4 inhibitor screening kits were purchased from Sigma-Aldrich (St. Louis, MO, USA). In addition, 3,5-dinitrosalicylic acid (DNS) was purchased from Beijing Solarbio Science & Technology Co., Ltd. (Beijing, China). A Total Antioxidant Capacity Assay Kit and Total Superoxide Dismutase Assay Kit were purchased from Beyotime Institute of Biotechnology (Shanghai, China). A Hydroxyl Free Radical assay kit was purchased from the Nanjing Jiancheng Bioengineering Institute (Nanjing, China). Antibiotic discs were purchased from Hangzhou Binhe Microorganism Reagent Co., Ltd. (Hangzhou, China). The HT-29 human colon adenocarcinoma cell line was purchased from the type culture cell bank of the Chinese Academy of Sciences (Shanghai, China).

### 2.3. Cell Free Supernatant (CFS), Intact Cell (IC) and Intracellular Cell Free Extraction (CFE) Preparation

The preparations of cell-free supernatant (CFS), intact cells (IC) and intracellular cell-free extract (CFE) were performed according to the method of Ragul et al. [15] with slight modifications.

LAB cultures were incubated in de Man Rogosa and Sharpe (MRS) broth (3% *v*/*v*) at 37 °C for 18 h and centrifugated at 3600× *g* for 10 min at 4 °C, and the collected supernatant was sterilized with 0.22 μm filter to obtain the cell-free supernatant (CFS).

The harvested cells were washed three times with sterile phosphate buffer solution (PBS, pH 7.4), then resuspended in PBS and adjusted to about 1×10^9^ CFU/mL to obtain the intact cell (IC).

For the preparation of intracellular cell free extract (CFE), cells were disrupted by VCX750 ultrasonic processor (Sonics&Material, Inc., Newtown, CT, USA) and performed at 5 s pulse on/3 s pulse off for 10 min in an ice bath. Cell fraction was removed by centrifugation (10,000× *g*, 20 min, 4 °C), and the supernatant was sterilized with 0.22 μm filter and kept at −20 °C.

### 2.4. Probiotic Property

#### 2.4.1. Tolerance to Artificial Gastric Juice

Artificial gastric juice contains 0.24 g/L of KH2PO4, 1.44 g/L of Na2HPO4, 8.00 g/L of NaCl, 0.20 g/L of KCl and 3.00 g/L of pepsin (adjust to pH 2.0). Cells were resuspended in artificial gastric juice to 109 CFU/mL and incubated at 37 °C for 2 h. The viable bacterial number was detected by plate colony counting method with MRS agar [20]. The MRS agar plate was incubated at 37 °C for 24 h. The survival rate was calculated according to the following formula.

Survival rate (%) = log CFU N/log CFU N_0_, (1)

N_0_: colony count of LAB before incubation; N: colony count of LAB after incubated 2 h.

#### 2.4.2. Tolerance to Artificial Bile Salt

The LAB strains (1%, *v*/*v*) were incubated in MRS-THIO broth (MRS broth supplemented with 0.20% sodium thioglycolate) with or without 0.30% (*w*/*v*) bile salt (oxgall) for 9 h or the absorbance reached 0.3 unit at 600 nm. The time required for the absorbance increased to 0.3 unit at 600 nm was calculated, and the time difference between two media was regarded as lag time (LT) [21].

#### 2.4.3. Cell Adhesion Activity

Auto aggregation, cell surface hydrophobicity and adhesion to HT-29 cells were tested to evaluate cell adhesion activity.

Auto aggregation of LAB was evaluated according to Vasiee et al.’s method with a modification [22]. Cells were collected by centrifugation at 3600× *g* for 10 min at 4 °C, washed twice with PBS (pH 7.4) and resuspended in PBS to OD_initial_ = 0.50 ± 0.05. Cell suspension was vortexed and incubated at 37 °C for 2 h. Absorbance at 600 nm of the suspension was measured (OD_final_).

Auto aggregation(%) = 100 × (OD_initial_ − OD_final_)/OD_initial_, (2)

The cell surface hydrophobicity of LAB was evaluated according to a modified method of Mohanty et al. [23]. Cells were resuspended in PBS (OD_initial_ = 0.7). In total, 3 mL cell suspension was mixed with 1 mL xylene and the mixture was rotated for 3 min and incubated at 37 °C for 60 min to separate two phases. The water phase was collected carefully and absorbance at 600 nm (OD_final_) was measured.

Cell surface hydrophobicity(%) = 100 × (OD_initial_ − OD_final_)/OD_initial_, (3)

HT-29 cells were grown in a high-glucose DMEM supplemented with 10% fetal bovine serum and 1% antibiotics (Penicillin-Streptomycin Solution) at 37 °C in a 5% CO_2_ atmosphere. The HT-29 cells were cultured in a 6-well cell culture plate for 48 h to obtain a monolayer of HT-29 cells. The LAB were resuspended in DMEM (without antibiotics and serum) and adjusted to 10^8^ CFU/mL to prepare an intact cell-DMEM suspension (IC-DMEM). The monolayers of HT-29 cells were washed three times with PBS, and then 1 mL IC-DMEM was added to each well. After incubating for 1 h, unattached bacteria were removed by washing three times with PBS. Then, the cells were fixed with 1 mL methanol, and an air-dry and Gram staining were performed after 30 min; the cells were observed under oil immersion. The number of LAB adhered to 100 HT-29 cells in 20 microscope fields was counted (5 cells were randomly selected from each microscope field). Non-adhesive was scored when less than 100 LAB cells adhered, medium adhesion when 100–1000 LAB cells adhered and strong adhesion when more than 1000 LAB cells adhered [24].

### 2.5. Hypoglycemic Ability

#### 2.5.1. Inhibition of α-Amylase

The inhibition of α-amylase was evaluated according to the method modified from Lee et al. [25]. The mixture contained 500 μL PBS (pH 7.4), 500 μL α-amylase solution (0.5 mg/mL) and 500 μL sample (CFS, IC or CFE). The mixture was incubated at 37 °C for 10 min, then 500 μL starch solution (1% *w*/*v*) was added and incubated at 37 °C for 3 min. Then, 1 mL DNS was added into the mixture, and was incubated in boiling water bath for 5 min, followed by the addition of 5 mL distilled water prior to cooling to room temperature. Absorbance was measured at 540 nm (acarbose was used as a positive control).

α-amylase inhibition rate(%) = 1 − [(A − B)/C],(4)

A: absorbance of the experimental sample (samples and α-amylase); B: absorbance of the blank (samples without α-amylase); C: absorbance of the control (with α-amylase and without samples).

#### 2.5.2. Inhibition of α-Glucosidase

According to the method of Yang et al. [26] with slight modification, the α-glucosidase inhibitory activity was evaluated. The reaction mixture contained 25μL sample, 50 μL glucosidase solution (0.2 U/mL) and 150 μL phosphate buffer solution (0.1 mol/L, pH 7.4). After pre-incubation, 75μL p-nitrophenyl-α-d-glucopyranoside (pNPG) solution (dissolved in phosphate buffer solution, 20 mmol/L) was added to the mixture and incubated at 37 °C for 10 min. The reaction was terminated by adding 1 mL 0.1 mol/L Na_2_CO_3_ solution. The amount of p-nitrophenol (pNP) was evaluated by measuring the absorbance at 405 nm. Acarbose was used as a positive control. The α-glucosidase inhibition rate was calculated according to the following formula:

α-glucosidase inhibition rate(%) = 1 − [(C − D)/(A − B)],(5)

A: with α-glucosidase and without samples; B: without α-glucosidase and samples; C: with samples and α-glucosidase; D: with samples but without α-glucosidase.

#### 2.5.3. Inhibition of DPP4

DPP4 enzyme inhibitory activity was evaluated using the DPP4 inhibitor screening kit by the method of the manufacturer (Sigma-Aldrich). The DPP4 enzyme cleaved the non-fluorescent substrate (H-Gly-Pro-AMC) and released the 7-amino-4-methyl coumarin (AMC) which was fluorescent. The fluorescence emissions (λex = 360 nm, λem = 460 nm) were collected in kinetic mode from 15 to 30 min on black clear-bottomed 96-well plates by SpectraMax i3X Microplate Reader (Molecular Devices China, Shanghai, China). The formula was used to calculate relative inhibition, where Δ*F*/Δ*T* represented the change in fluorescence concerning the chosen time interval:

(6) $$\mathrm{Relative}\;\mathrm{Inhibition}\left(\%\right)=\frac{\frac{\Delta F}{\Delta T}\mathrm{Enzyme}-\frac{\Delta F}{\Delta T}\mathrm{Enzyme}\;\mathrm{Inhibitor}\;\mathrm{complex}}{\frac{\Delta F}{\Delta T}\mathrm{Enzyme}}\times 100,$$

### 2.6. Antioxidant Activity

#### 2.6.1. DPPH Radical Scavenging Activity

DPPH radical scavenging activity was measured by the method of Jung et al. [27] with a slight modification. Briefly, 500 μL sample was mixed with 500 μL DPPH solution (100 μmol/L), and the reaction was carried out at 25 °C in a dark condition. After 30 min, the mixture was centrifugated at 6000× *g* for 10 min and the absorbance of the supernatant was measured at 517 nm by a UV–Visible spectrophotometer (Beijing Persee General Instrument Co., Ltd., Beijing, China). DPPH radical scavenging activity was calculated as follows:

(7) $$\mathrm{DPPH}\;\mathrm{radical}\;\mathrm{scavenging}\;\mathrm{activity}\;\left(\%\right)=\left(1-\frac{A-B}{C}\right)\times 100,$$

A: absorbance of sample; B: absorbance of the blank group; C: absorbance of the control group.

#### 2.6.2. Hydroxyl Radical Scavenging Activity

The hydroxyl radical (·OH) scavenging activity was investigated by a Hydroxyl Free Radical assay kit. The amount of H_2_O_2_ is directly proportional to the amount of OH produced by the Fenton reaction. When the electron acceptor is given, it is colored with the Griess reagent to form a red substance, and its color is positively proportional to the amount of OH. The reaction mixture contained 0.2 mL samples, 0.2 mL substrate application solution and 0.4 mL application solution. The mixture added with the chromogenic agent was placed at room temperature for 20 min, and then the absorbance at 550 nm was measured. The hydroxyl radical scavenging activity was calculated according to the following formula.

(8) $$\mathrm{Hydroxyl}\;\mathrm{radical}\;\mathrm{scavenging}\;\mathrm{activity}\;\left(\%\right)=\left(1-\frac{A-B}{C}\right)\times 100$$

A: absorbance of sample; B: absorbance of the blank group (without sample and substrate application solution instead of double distilled water); C: absorbance of the control group (without sample and instead of double distilled water).

#### 2.6.3. Superoxide Dismutase Activity

The superoxide dismutase (SOD) activity was investigated by the Total Superoxide Dismutase Assay Kit according to the manufacturer’s instruction. The SOD catalyzed the disproportionation of superoxide anion (O_2_^−^); the reaction between WST-8 and superoxide anion catalyzed by xanthine oxidase to produce the formazan dye (absorbance at 450 nm), and the result was expressed as the inhibition rate. The reaction mixture contained a 20 μL sample, 160 μL WST-8/enzyme working solution and 20 μL reaction starting working solution. After incubation at 37 °C for 30 min, the absorbance was measured at 450 nm.

(9) $$\mathrm{Inhibiton}\;\mathrm{rate}\;\left(\%\right)=\frac{\left(B-C\right)-\left(A-D\right))}{B-C}\times 100$$

A: absorbance of sample; B: absorbance of the blank group (without sample); C: absorbance of the control group (without sample and reaction starting working solution); D: absorbance of the control group (without reaction starting working solution).

#### 2.6.4. Ferric-Reducing Antioxidant Power (FRAP)

The ferric reducing antioxidant power (FRAP) assay was based on the activity of antioxidants to reduce Fe^3+ ^into Fe^2+^ in the presence of tripyridyltriazine (TPTZ), forming an intense blue Fe^2+^–TPTZ complex with the absorption maximum at 593 nm [28]. The FRAP assay was performed using the Total Antioxidant Capacity Assay Kit with the FRAP method. The 5 μL sample was mixed with 180 μL FRAP working solution and kept at 37 °C for 5 min. The absorbance of the reaction mixture was recorded at 593 nm. The standard curve was prepared using FeSO_4_ in the range of 0.15 to 1.5 mmol/L. The amount of FeSO_4_ equivalent to the sample was used to express the antioxidant activity of the sample.

### 2.7. Antibiotic Susceptibility

The antibiotic susceptibility was evaluated by the disc diffusion method [29]. Eight kind of antibiotics susceptibility discs contained kanamycin (30 µg), gentamicin (10 µg), tetracycline (30 µg), chloramphenicol (30 µg), streptomycin (25 µg), ampicillin (10 µg), erythromycin (15 µg) and penicillin (10IU), respectively. The LAB suspension was diluted to 1.5 × 10^8^ CFU/mL and coated on the entire surface of the MRS plate. The antibiotic susceptibility discs were placed on the surface of the MRS plate and incubated at 37 °C for 24 h. The diameter of the growth inhibition zone (mm) was noted.

### 2.8. Statistics Analysis

All experiments were repeated three times and data were shown as means ± standard deviation. The statistical analysis was completed using SPSS 22.0 (SPSS Inc., Chicago, IL, USA). Statistical significances were analyzed using a one-way analysis of variance (ANOVA) followed by Tukey’s HSD multiple comparison tests (*p* < 0.05).

## 3. Result and Discussion

### 3.1. Tolerance to Artificial Gastric Juice and Bile Salt

#### 3.1.1. Tolerance to Artificial Gastric Juice

**Figure 1:**
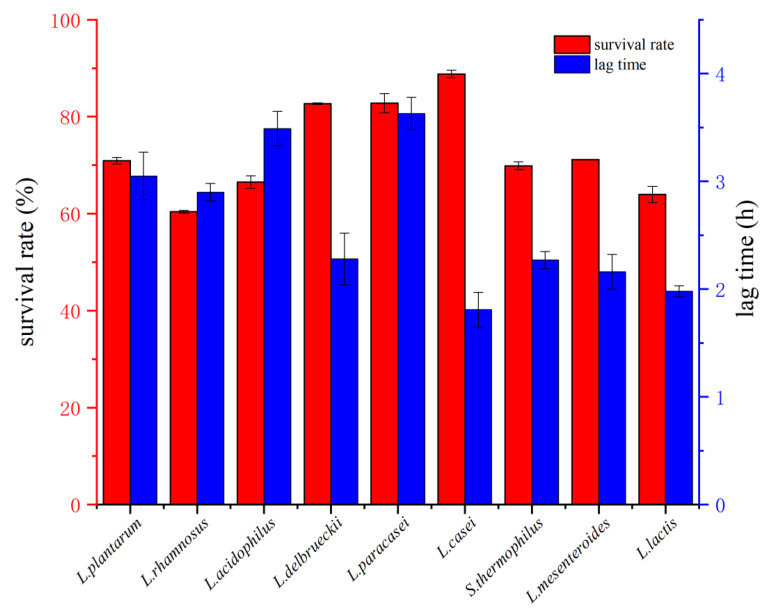
Tolerance to artificial gastric juice and bile salt of LAB.

LAB are inhibited or even killed in gastric juice [30]. The tolerance of LAB to gastric juice was measured by the survival rate of LAB after incubation in simulated gastric juice. The results are shown in Figure 1. After incubation for 2 h, the survival rate of LAB ranged from 60.40% to 88.81%. *L. casei* had the highest survival rate. *L. paracasei* (82.78%) and *L. delbrueckii* (82.68%) also had higher survival rates than *L. plantarum* AM3 (81.1%) and *L. plantarum* NG13 (82.0%), as reported by Fadare OS et al. [31]; however, *L. plantarum*(70.91%) in this study was lower than *L. plantarum* AM3 and *L. plantarum* NG13. This result may be caused by the difference of cell size of LAB, because long strains had the worst tolerance to artificial gastric juice [32].

#### 3.1.2. Tolerance to Artificial Bile Salt

Tolerance to bile salt can be used to evaluate the viability of LAB in the intestine. LAB can maintain cell membrane by activating surface proteins, so as to resist bile salts [33]. As shown in Figure 1, the tolerance to bile salt was expressed by lag time. The lag time of nine kinds of LAB ranged from 1.81 h to 3.63 h. *L. casei* (1.81 h) showed the shortest lag time. *L. lactis* (1.98 h) and *L. mesenteroides* (2.16 h) had lower lag times than *L. lactis* 100 (6.35 h) and *L. mesenteroides* 153 (4.61 h) in a previous study [34]. Chen et al. [35] reported the lag time of *S. thermophilus* S28, *S. thermophilus* 200711y1 and *L. plantarum* S3 were more than 24 h, which was much higher than LAB in this study. In addition, the bile tolerances of *L. acidophilus* (3.49 h) and *L. plantarum* (3.05 h) were better than those of *L. acidophilus* AD1 (>24 h) and *L. plantarum* KLDS1.0391 (5.75 h) in the research of Han et al. [36]. Research showed that the lag time of LAB may be lower than 0.20 h (0.14 h for *S. thermophilus* 129, 0.16 h for *L. plantarum* 140) or higher than 24 h (*S. thermophilus* S28, *S. thermophilus* 200711y1, *L. plantarums* 3 and *L. acidophilus* AD1) [34,35,36]. The lactic acid bacteria in this study had relatively good tolerance to bile salt. The difference between LTs of LAB may be due to the ability to maintain the cell membrane by activating surface proteins.

### 3.2. Cell Adhesion Ability

#### 3.2.1. Auto Aggregation and Cell Surface Hydrophobicity

**Figure 2:**
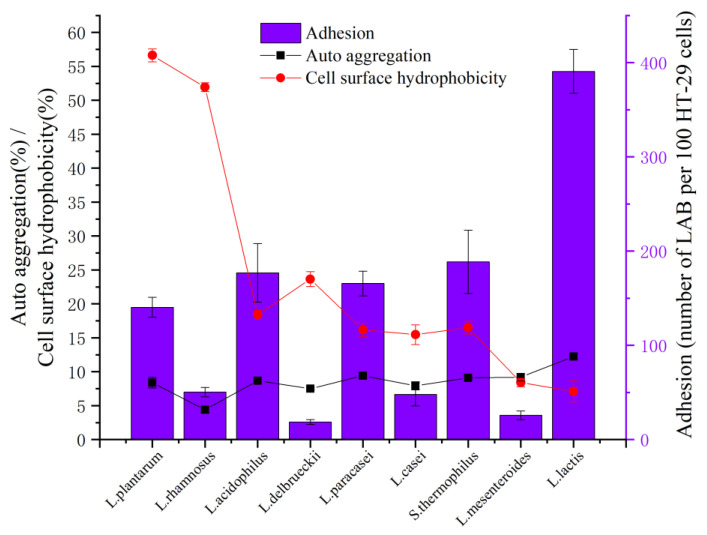
Cell adhesion ability of LAB.

Auto aggregation and cell surface hydrophobicity are directly related to the adhesion ability of probiotics, which make them better adhere to the intestinal surface and form biofilm [37,38,39]. The results of auto aggregation and cell surface hydrophobicity are shown in Figure 2.

The absorbance of bacterial suspension decreased (4.38% to 12.23%) due to auto aggregation; this result was consistent with the report of Zhao et al. [40]. The auto aggregation ability of *L. lactis* (12.23%) was significantly higher than other strains (*p* < 0.05). The results showed that the cell surface hydrophobicity of LAB had great differences (ranged from 7.11% to 56.63%), as in the result of Abushelaibi et al. [41], which ranged from 2.7% to 67.0%. *L. plantarum* (56.63%) and *L. rhamnosus* (51.94%) had higher cell surface hydrophobicity than other strains. The surface hydrophobicity of LAB may be related to the glycoproteins on the cell surface [42]. In this study, the auto aggregation activity of *Lactobacillus* was lower or had no significant difference compared with other strains (*p* < 0.05), but the hydrophobicity of LAB had the opposite trend (Figure 2).

#### 3.2.2. HT-29 Cell Adhesion Activity

The adhesion activity of HT-29 cells in vitro is an important index to evaluate the probiotic characteristics of LAB. The HT-29 cell adhesion activity was shown in Figure 2. The highest adhesion index was *L. lactis* (390.67/100 cell), and the lowest was *L. delbrueckii* (18.67/100 cell). *L. plantarum*, *L. acidophilus*, *L. paracasei*, *S. thermophilus* and *L. lactis* had medium adhesion activity (>100 bacteria/100 cell). However, *L. rhamnosus*, *L. delbrueckii*, *L. casei* and *L. mesenteroides* showed non-adhesiveness (<100 bacteria/100 cell).

### 3.3. Hypoglycemic Ability

#### 3.3.1. Inhibition of α-Amylase

The inhibition of α-amylase activity may help to reduce postprandial blood glucose rise. As shown in Table 1, all CFS showed good inhibition of α-amylase activity (62.28%–83.36%). *L. plantarum*, *L. delbrueckii, L. paracasei* and *L. casei* had significantly higher inhibition activity (*p* < 0.05), and *L. plantarum* had the highest inhibition rate (83.36%). The inhibitory activities of *L. lactis* and *L. mesenteroides* were higher than *S. thermophilus.* The results showed that the α-amylase inhibitory activity of LAB mainly depended on the CFS (fermentation products); however, IC and CFE (intracellular substances) showed little α-amylase inhibitory activity. It is similar to the result reported by Lee et al. [25]. Among all LAB, *L. plantarum* (83.36%), *L. delbrueckii* (82.83%), *L. paracasei* (82.21%), *L. casei* (79.27%) and *L. lactis* (78.83%) had better inhibitory activity, and their inhibitory activity on α-amylase were higher than 1 mg/mL acarbose (78.65%).

#### 3.3.2. Inhibition of α-Glucosidase

α-glucosidase located in the intestine could hydrolyze a variety of sugars to glucose, and the inhibition of α-glucosidase could reduce the blood glucose level [43]. α-glucosidase inhibitory activities of LAB were shown in Table 1. In this study, inhibitory activities were detected in the CFS, IC and CFE of LAB, but the CFE was lower. The CFS of *L. plantarum* (85.16%) had shown the highest α-glucosidase inhibitory activity, while the α-glucosidase inhibitory activity of the IC of *L. paracasei* (84.29%) was higher than other strains. The highest (*p* < 0.05) α-glucosidase inhibitory activity of the CFE was founded in *L. lactis* (29.17%), *L. casei* (28.78%) and *L. paracasei* (26.17%). In a previous study, the α-glucosidase inhibitory activities of the CFS of *L. plantarum* T34 (10.24%) and *L. rhamnosus* GG (14.58%) were determined, which were lower than the LAB in this study [44].

#### 3.3.3. Inhibition of Dipeptidyl Peptidase 4

Dipeptidyl peptidase 4 (DPP4) is a new target for the treatment of type 2 diabetes and plays an important role in the immune system, nervous system and endocrine system. DPP4 can hydrolyze glucagon like peptide-1 (GLP-1), resulting in the increase in blood glucose [45]. In this study, DPP4 inhibitory activity was not found in the CFS of LAB (Table 1). Comprehensively, *L. paracasei* had the best DPP4 inhibitory activity, and its IC (42.51%) and CFE (32.66%) had the highest DPP4 inhibitory rate (*p* < 0.05); this result is higher than *L. paracasei* KLDS 1.0351 (8.8%) reported by Yan et al. [44]. Zhang et al. [5] found that the inhibition of DPP4 may be related to some functional peptides produced by LAB. The higher DPP4 inhibitory activity of *L. paracasei* may be due to the production of more DPP4 inhibitory peptides. Furthermore, this may be because the gene coding peptides that inhibited DPP4 had higher expressions, or enzymes involved in related pathways had higher activity, but its real mechanism and effect need to be further studied.

### 3.4. Antioxidant Activity

#### 3.4.1. DPPH Radical Scavenging Activity

The scavenging activity of the 2,2-diphenyl-1-picrylhydrazyl (DPPH) radical could be used to evaluate antioxidant capacity [46]. The DPPH radical scavenging activity of the CFS, IC and CFE of LAB is shown in Table 2. The results show that the DPPH radical scavenging rates of the CFS were higher than CFE and IC; this result was consistent with *L. plantarum* MA2 tested by Tang et al. [47]. The scavenging rates of the CFS of *L. rhamnosus* (73.02%) and *L. plantarum* (71.37%) were significantly (*p* < 0.05) higher than other samples. *L. mesenteroides* showed better scavenging activity of the DPPH radical than *S. thermophilus* and *L. lactis.*

#### 3.4.2. Total Antioxidant Activity (FRAP)

Total antioxidant activity measured by ferric-reducing antioxidant power (FRAP) was shown in Table 2. The CFE and CFS of LAB were detected to have total antioxidant activity, but the IC was not detected. The CFS of *L. plantarum* (1.19 FeSO_4_·7H_2_O eq mmol/L) and *L. acidophilus* (1.21 FeSO_4_·7H_2_O eq mmol/L) showed higher FRAP activity. The CFE of strains, except *L. paracasei*, had significantly higher total antioxidant activity (*p* < 0.05).

#### 3.4.3. Hydroxyl Radical Scavenging Activity

Hydroxyl radical (·OH) is a reactive oxygen species, which can lead to DNA damage and lipid peroxidation [48]. In this study, hydroxyl radical scavenging activity was detected (Table 2). The hydroxyl radical scavenging rate of the IC was lower than that of the CFE and CFS. Among the CFS, IC and CFE, of all strains, the highest hydroxyl radical scavenging rates were *L. acidophilus* (64.81%), *L. lactis* (30.58%) and *L. casei* (50.69%), respectively. The hydroxyl radical scavenging rate of *L. rhamnosus* (21.07%) in the IC was similar to *L. rhamnosus* (23.55%) in a previous study [44].

#### 3.4.4. SOD Activity

Superoxide dismutase (SOD) activity plays an important role in alleviating oxidative stress [49]. The results of SOD activity were shown in Table 2. The SOD activity of CFS was higher than CFE, but could not be detected in the IC. The CFS of *L. plantarum* (35.01%) and *L. acidophilus* (33.35%) had the highest SOD activity. The CFE of *L. acidophilus* (23.45%), *L. plantarum* (22.27%) and *L. casei* (22.09%) had the best SOD activity. According to the results of the CFS and CFE, *L. plantarum* and *L. acidophilus* may have had the best superoxide dismutase activity among all the strains. (*p* < 0.05)

### 3.5. Antibiotics Susceptibility

Studies had shown that probiotics with antibiotic resistance may transfer related antibiotic resistance genes to other intestinal bacteria [50]. Therefore, in order to evaluate the safety of lactic acid bacteria, antibiotic susceptibility was tested. The results (Table 3) showed that all LAB were sensitive to four common antibiotics (ampicillin, chloramphenicol, erythromycin and tetracycline). In addition, *L. plantarum*, *L. rhamnosus*, *L. acidophilus*, *L. delbrueckii*, *L. paracasei*, *S. thermophilus* and *L. mesenteroides* were sensitive to penicillin G, and *S. thermophilus* and *L. mesenteroides* were sensitive to streptomycin. Some LAB in this study showed resistance to gentamicin, kanamycin and streptomycin. However, according to the previous studies [34], some LAB had instinct or natural resistance to some antibiotics (such as gentamicin, kanamycin and streptomycin), and this resistance gene is non-transmissible. LAB resistant to gentamicin, kanamycin and streptomycin are not necessarily unsafe, and further studies are needed to verify whether resistance genes of LAB were non-transmissible.

### 3.6. Principal Component Analysis (PCA) and Analytic Hierarchy Process (AHP)

A principal component analysis (PCA), which focuses on replacing multiple possibly related variables with less uncorrelated comprehensive variables, is a very useful analysis method in screening functional probiotics [35]. The analytic hierarchy process (AHP) is a method to divide a complex problem into several orderly levels, and then analyze and compare these levels. The two methods determine the weight of factors in different ways (PCA is an objective weight method, AHP is a subjective weight method). There are many factors that need to be considered in the screening of hypoglycemic probiotics; therefore, the combination of PCA and AHP was used for screening of hypoglycemic LAB in this study.

**Figure 3:**
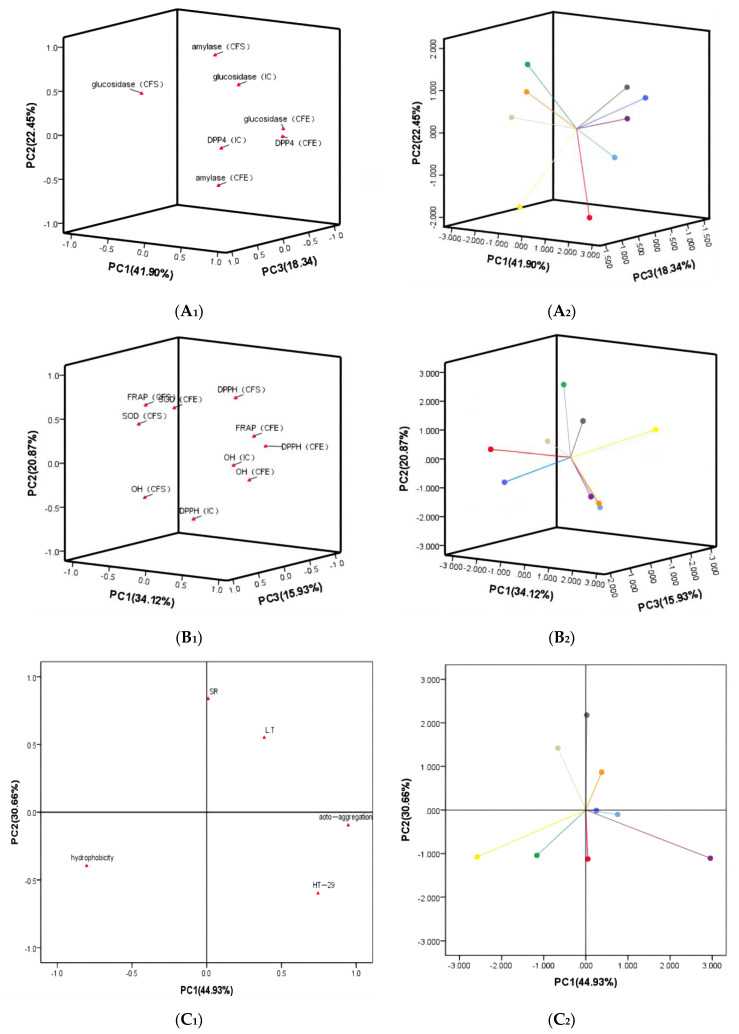
Comprehensive evaluation of LAB by principal component analysis. A: Hypoglycemic ability; B: Antioxidant activity; C: Probiotic property. (**A_1_**,**B_1_**,**C_1_**) represent factor loading; (**A_2_**,**B_2_**,**C_2_**) represent factor scores of *L. plantarum* (*●*), *L. rhamnosus* (*●*), *L. acidophilus* (*●*), *L. delbrueckii* (*●*), *L. paracasei* (*●*), *L. casei* (*●*), *S. thermophilus* (*●*), *L. mesenteroides* (*●*), *L. lactis* (*●*).

The indicators in the PCA were divided into probiotic property, hypoglycemic ability and antioxidant activity. Antibiotic susceptibility was used to evaluate the safety of lactic acid bacteria, so it was not included in the PCA. Results of the PCA are shown in Figure 3. Three independent principal components (PCs) of the hypoglycemic ability were extracted. As shown in Figure 3A, PC1 accounted for 41.90% of the total variance, PC2 accounted for 22.45% and PC3 accounted for 18.34%. Four principal components were extracted from the PCA of antioxidant activity: PC1 accounted for 34.12%, PC2 accounted for 20.87%, PC3 accounted for 15.93% and PC4 accounted for 11.80% (only PC1, PC2 and PC3 are included in Figure 3B). Two PCs were extracted from the PCA of probiotic properties (Figure 3C): PC1 accounted for 44.93% and PC2 accounted for 30.66%. According to the scores of the PCA, *L. rhamnosus* (0.948) had the best antioxidant activity, *L. paracasei* (1.555) had the best hypoglycemic ability and *L. lactis* (1.308) had the highest probiotic property.

In the AHP, hypoglycemic ability, antioxidant activity and probiotic property were compared through Santy’s scaling method and the judgment matrix was established (hypoglycemic ability: antioxidant activity = 2:1; hypoglycemic ability: probiotic property = 3:1; antioxidant activity: probiotic property = 2:1). The weights of hypoglycemic ability, antioxidant activity and probiotic property were 53.896%, 29.726% and 16.378%, respectively. In further calculation, the consistency ratio (CR) was 0.009, which showed that the judgment matrix met the consistency test, and the calculated weights were consistent (CR < 0.1). According to the comprehensive score of the PCA with weight, *L. paracasei* (0.657) was the best potential hypoglycemic probiotic, followed by *L. acidophilus* (0.387) and *L. plantarum* (0.256). Additionally, *L. lactis* (0.160) showed a higher comprehensive score than *L.mesenteroides* and *S. thermophilus*.

## 4. Conclusions

In this study, the hypoglycemic ability, antioxidant activity and probiotic property of LAB were comprehensively analyzed by combining the analytic hierarchy process (AHP) and principal component analysis (PCA). The results showed that *L. paracasei*, *L. rhamnosus* and *L. lactis* had the best comprehensive score in hypoglycemic ability, antioxidant activity and probiotic property, respectively. According to the comprehensive score of the PCA and AHP, *L. paracasei* showed very prominent hypoglycemic potential, and *L. lactis* showed a higher comprehensive score than *L.mesenteroides* and *S. thermophilus*. All strains showed good safety in antibiotic susceptibility and can be used as probiotics for food. In the future, the verification of the hypoglycemic potential of the strains *in vivo* experiments, and the molecular mechanism of hypoglycemic ability, are needed before commercial exploitation.

## Figures and Tables

**Table 1:** Hypoglycemic ability of LAB.

Hypoglycemic Ability	Strain	*L. plantarum*	*L. rhamnosus*	*L. acidophilus*	*L. delbrueckii*	*L. paracasei*	*L. casei*	*S. thermophilus*	*L. mesenteroides*	*L. lactis*	Acarbose
α-amylase inhibitory activities(%)	CFS	83.36 ± 0.77 ^a^	62.28 ± 0.67 ^e^	72.51 ± 1.73 ^d^	82.83 ± 0.55 ^ab^	82.21 ± 1.12 ^abc^	79.27 ± 1.45 ^abc^	71.09 ± 1.81 ^d^	78.47 ± 0.88 ^c^	78.83 ± 0.77 ^bc^	78.65 ± 1.78
	IC	ND	ND	ND	ND	ND	ND	ND	ND	ND	
	CFE	ND	ND	7.77 ± 0.21	ND	ND	ND	ND	ND	ND	
α-glucosidase inhibitory activities (%)	CFS	85.16 ± 0.32 ^a^	82.55 ± 1.76 ^a^	29.04 ± 2.79 ^e^	56.90 ± 1.64 ^c^	44.53 ± 2.49 ^d^	69.66 ± 1.21 ^b^	52.86 ± 2.67 ^c^	83.20 ± 1.15 ^a^	51.95 ± 3.68 ^cd^	91.90 ± 2.94
	IC	83.81 ± 1.78 ^a^	73.81 ± 5.26 ^c^	82.86 ± 2.33 ^ab^	78.10 ± 3.37 ^bc^	84.29 ± 1.17 ^a^	81.90 ± 3.56 ^a^	78.10 ± 1.78 ^abc^	81.43 ± 1.17 ^abc^	78.57 ± 2.33 ^abc^	
	CFE	5.21 ± 1.44 ^d^	9.38 ± 1.69 ^cd^	17.84 ± 1.61 ^b^	4.56 ± 2.08 ^d^	26.17 ± 1.94 ^a^	28.78 ± 1.81 ^a^	16.15 ± 2.26 ^bc^	8.07 ± 1.61 ^d^	29.17 ± 3.81 ^a^	
DPP4 inhibitory activities (%)	CFS	ND	ND	ND	ND	ND	ND	ND	ND	ND	
	IC	14.91 ± 1.84 ^d^	7.22 ± 2.01 ^e^	27.30 ± 1.58 ^b^	ND	42.51 ± 1.25 ^a^	25.55 ± 1.61 ^bc^	36.69 ± 1.37 ^a^	6.13 ± 2.04 ^e^	20.61 ± 1.72 ^cd^	
	CFE	11.31 ± 5.43 ^b^	10.56 ± 5.48 ^b^	23.44 ± 4.69 ^ab^	12.83 ± 5.34 ^b^	32.66 ± 4.12 ^a^	ND	ND	ND	ND	

Note: ND: not detected; CFS: Cell-free supernatant; IC: Intact cell; CFE: Cell-free extracts. Different letters in same column indicate significant differences (*p* < 0.05).

**Table 2:** Antioxidant activities of LAB.

Antioxidant activities	Strain	*L. plantarum*	*L. rhamnosus*	*L. acidophilus*	*L. delbrueckii*	*L. paracasei*	*L. casei*	*S. thermophilus*	*L. mesenteroides*	*L. lactis*
Scavenging rate of DPPH radical (%)	CFS	71.37 ± 0.53 ^a^	73.02 ± 0.24 ^a^	60.08 ± 0.81 ^e^	66.01 ± 0.45 ^bc^	62.56 ± 0.30 ^d^	64.64 ± 0.40 ^c^	57.43 ± 0.29 ^f^	67.28 ± 0.11 ^b^	59.04 ± 0.67 ^ef^
	IC	8.15 ± 1.71 ^de^	6.06 ± 0.31 ^ef^	11.81 ± 0.66 ^c^	7.13 ± 0.56 ^e^	9.77 ± 0.42 ^cd^	4.50 ± 0.25 ^f^	9.77 ± 0.59 ^cd^	19.66 ± 0.08 ^a^	15.41 ± 0.37 ^b^
	CFE	18.46 ± 2.33 ^b^	26.37 ± 1.42 ^a^	10.65 ± 1.66 ^c^	11.08 ± 1.61 ^c^	8.44 ± 1.76 ^c^	8.23 ± 2.11 ^c^	14.35 ± 2.15 ^bc^	19.62 ± 2.37 ^ab^	14.35 ± 1.72 ^bc^
Total antioxidant activity(FeS_O4_·7H_2_O eq mmol/L)	CFS	1.19 ± 0.06 ^a^	1.00 ± 0.04 ^c^	1.21 ± 0.05 ^a^	1.14 ± 0.01 ^ab^	1.15 ± 0.01 ^ab^	1.15 ± 0.02 ^ab^	0.98 ± 0.02 ^cd^	0.85 ± 0.03 ^d^	1.06 ± 0.04 ^bc^
	IC	ND	ND	ND	ND	ND	ND	ND	ND	ND
	CFE	1.25 ± 0.01 ^a^	1.24 ± 0.05 ^a^	1.19 ± 0.02 ^a^	1.28 ± 0.05 ^a^	1.03 ± 0.01 ^b^	1.30 ± 0.06 ^a^	1.28 ± 0.01 ^a^	1.19 ± 0.02 ^a^	1.29 ± 0.02 ^a^
Hydroxyl radical scavenging activity(%)	CFS	41.12 ± 0.51 ^e^	39.12 ± 0.49 ^f^	64.81 ± 0.10 ^a^	52.96 ± 0.26 ^c^	60.40 ± 0.26 ^b^	49.86 ± 0.68 ^d^	61.43 ± 0.42 ^b^	41.74 ± 0.44 ^e^	51.79 ± 1.09 ^c^
	IC	22.24 ± 1.12 ^b^	21.07 ± 0.94 ^b^	13.09 ± 0.39 ^cd^	8.75 ± 0.49 ^e^	14.05 ± 0.51 ^c^	11.43 ± 0.52 ^d^	11.09 ± 0.83 ^de^	21.01 ± 0.19 ^b^	30.58 ± 0.73 ^a^
	CFE	45.45 ± 0.77 ^b^	45.73 ± 0.83 ^b^	41.94 ± 0.45 ^c^	40.91 ± 0.51 ^c^	45.94 ± 0.49 ^b^	50.69 ± 0.85 ^a^	50.14 ± 0.35 ^a^	46.97 ± 0.35 ^b^	46.49 ± 0.58 ^b^
SOD activity (%)	CFS	35.01 ± 0.78 ^a^	28.60 ± 1.56 ^e^	33.35 ± 0.61 ^ab^	32.36 ± 1.18 ^bc^	32.75 ± 0.25 ^b^	32.00 ± 0.26 ^bcd^	29.91 ± 0.25 ^de^	32.59 ± 0.96 ^bc^	30.42 ± 0.85 ^cde^
	IC	ND	ND	ND	ND	ND	ND	ND	ND	ND
	CFE	22.27 ± 0.3 ^a^	17.53 ± 0.36 ^b^	23.45 ± 0.57 ^a^	15.57 ± 0.84 ^b^	17.30 ± 1.23 ^b^	22.09 ± 1.81 ^a^	17.62 ± 0.67 ^b^	15.85 ± 1.06 ^b^	18.03 ± 0.39 ^b^

Note: ND: Not detected; CFS: Cell-free supernatant; IC: Intact cell; CFE: Cell-free extracts. Different letters in same column indicate significant differences (*p* < 0.05).

**Table 3:** Antibiotic susceptibility.

Strain	AMP	CHL	ERY	TET	PEN	STR	GEN	KAN
*L. plantarum*	*S*	*S*	*S*	*S*	*S*	*R*	*R*	*R*
*L. rhamnosus*	*S*	*S*	*S*	*S*	*S*	*R*	*R*	*R*
*L. acidophilus*	*S*	*S*	*S*	*S*	*S*	*R*	*R*	*R*
*L. delbrueckii*	*S*	*S*	*S*	*S*	*S*	*R*	*I*	*R*
*L. paracasei*	*S*	*S*	*S*	*S*	*S*	*R*	*R*	*R*
*L. casei*	*S*	*S*	*S*	*S*	*R*	*R*	*R*	*R*
*S. thermophilus*	*S*	*S*	*S*	*S*	*S*	*S*	*R*	*R*
*L. mesenteroides*	*S*	*S*	*S*	*S*	*S*	*S*	*R*	*R*
*L. lactis*	*S*	*S*	*S*	*S*	*R*	*R*	*R*	*R*

Note: S: Susceptible (Red); I: Intermediate (Yellow); R: Resistance (Blue); AMP: Ampicillin; CHL: Chloramphenicol; ERY: Erythromycin; TET: Tetracycline; PEN: Penicillin; STR: Streptomycin; GEN: Gentamicin; KAN: Kanamyci.

## Data Availability

The data presented in this study are available within the article.

## References

[1] Ogurtsova K., Da Rocha Fernandes J.D., Huang Y., Linnenkamp U., Guariguata L., Cho N.H., Cavan D., Shaw J.E., Makaroff L.E. (2017). IDF Diabetes Atlas: Global estimates for the prevalence of diabetes for 2015 and 2040. Diabetes Res. Clin. Pract..

[2] American Diabetes Association (2019). Classification and Diagnosis of Diabetes: *Standards of Medical Care in Diabetes—2019*. Diabetes Care.

[3] Han K., Park J.B. (2018). Clinical implication of fasting glucose and systolic/diastolic blood pressure on the prevalence of periodontitis in non-diabetic and non-hypertensive adults using nationally representative data. Exp. Ther. Med..

[4] Islam M.S. (2013). Animal Models of Diabetic Neuropathy: Progress Since 1960s. J. Diabetes Res..

[5] Zhang Z., Lv L. (2015). Effect of local insulin injection on wound vascularization in patients with diabetic foot ulcer. Exp. Ther. Med..

[6] Broichhagen J., Schönberger M., Cork S., Frank J.A., Marchetti P., Bugliani M., Shapiro A.M.J., Trapp S., Rutter G., Hodson D.J. (2014). Optical control of insulin release using a photoswitchable sulfonylurea. Nat. Commun..

[7] Oboh G., Ogunsuyi O.B., Ogunbadejo M.D., Adefegha S.A. (2016). Influence of gallic acid on α-amylase and α-glucosidase inhibitory properties of acarbose. J. Food Drug Anal..

[8] Tangvarasittichai S. (2015). Oxidative stress, insulin resistance, dyslipidemia and type 2 diabetes mellitus. World J. Diabetes.

[9] Phoem A.N., Mayiding A., Saedeh F., Permpoonpattana P. (2019). Evaluation of *Lactobacillus plantarum* encapsulated with Eleutherine americana oligosaccharide extract as food additive in yoghurt. Braz. J. Microbiol..

[10] Niibo M., Shirouchi B., Umegatani M., Morita Y., Ogawa A., Sakai F., Kadooka Y., Sato M. (2019). Probiotic *Lactobacillus gasseri* SBT2055 improves insulin secretion in a diabetic rat model. J. Dairy Sci..

[11] Toejing P., Khat-Udomkiri N., Intakhad J., Sirilun S., Chaiyasut C., Lailerd N. (2020). Putative Mechanisms Responsible for the Antihyperglycemic Action of *Lactobacillus paracasei* HII01 in Experimental Type 2 Diabetic Rats. Nutrients.

[12] Yan F., Li N., Shi J., Li H., Yue Y., Jiao W., Wang N., Song Y., Huo G., Li B. (2019). *Lactobacillus acidophilus* alleviates type 2 diabetes by regulating hepatic glucose, lipid metabolism and gut microbiota in mice. Food Funct..

[13] Zeng Z., Yuan Q., Yu R., Zhang J., Ma H., Chen S. (2019). Ameliorative Effects of Probiotic *Lactobacillus paracasei* NL41 on Insulin Sensitivity, Oxidative Stress, and Beta-Cell Function in a Type 2 Diabetes Mellitus Rat Model. Mol. Nutr. Food Res..

[14] Obaroakpo J.U., Liu L., Zhang S., Lu J., Pang X., Lv J. (2019). α-Glucosidase and ACE dual inhibitory protein hydrolysates and peptide fractions of sprouted quinoa yoghurt beverages inoculated with *Lactobacillus casei*. Food Chem..

[15] Ragul K., Kandasamy S., Devi P.B., Shetty P.H. (2020). Evaluation of functional properties of potential probiotic isolates from fermented brine pickle. Food Chem..

[16] Yan F., Li N., Yue Y., Wang C., Zhao L., Evivie S., Li B., Huo G. (2020). Screening for Potential Novel Probiotics With Dipeptidyl Peptidase IV-Inhibiting Activity for Type 2 Diabetes Attenuation in vitro and in vivo. Front. Microbiol..

[17] Zeng Z., Luo J., Zuo F., Zhang Y., Ma H., Chen S. (2016). Screening for potential novel probiotic *Lactobacillus* strains based on high dipeptidyl peptidase IV and α-glucosidase inhibitory activity. J. Funct. Foods.

[18] Walton E.L. (2017). Oxidative stress and diabetes: Glucose response in the cROSsfire. Biomed. J..

[19] Umeno A., Horie M., Murotomi K., Nakajima Y., Yoshida Y. (2016). Antioxidative and Antidiabetic Effects of Natural Polyphenols and Isoflavones. Molecules.

[20] Son S.-H., Jeon H.-L., Jeon E.B., Lee N.-K., Park Y.-S., Kang D.-K., Paik H.-D. (2017). Potential probiotic *Lactobacillus plantarum* Ln4 from kimchi: Evaluation of β-galactosidase and antioxidant activities. LWT.

[21] Liu W., Chen M., Duo L., Wang J., Guo S., Sun H., Menghe B., Zhang H. (2020). Characterization of potentially probiotic lactic acid bacteria and bifidobacteria isolated from human colostrum. J. Dairy Sci..

[22] Vasiee A., Falah F., Behbahani B.A., Tabatabaee-Yazdi F. (2020). Probiotic characterization of *Pediococcus* strains isolated from Iranian cereal-dairy fermented product: Interaction with pathogenic bacteria and the enteric cell line Caco-2. J. Biosci. Bioeng..

[23] Mohanty D., Panda S., Kumar S., Ray P. (2019). In vitro evaluation of adherence and anti-infective property of probiotic *Lactobacillus plantarum* DM 69 against Salmonella enterica. Microb. Pathog..

[24] Mohamad N., Manan H., Sallehhuddin M., Musa N., Ikhwanuddin M. (2020). Screening of Lactic Acid Bacteria isolated from giant freshwater prawn (*Macrobrachium rosenbergii*) as potential probiotics. Aquac. Rep..

[25] Lee S., Kim M. (2019). *Leuconostoc mesenteroides* MKSR isolated from kimchi possesses α-glucosidase inhibitory activity, antioxidant activity, and cholesterol-lowering effects. LWT.

[26] Yang X., Ren Y., Li L. (2022). The relationship between charge intensity and bioactivities/processing characteristics of exopolysaccharides from lactic acid bacteria. LWT.

[27] Jung J., Jang H.J., Eom S.J., Choi N.S., Lee N.-K., Paik H.-D. (2017). Fermentation of red ginseng extract by the probiotic *Lactobacillus plantarum* KCCM 11613P: Ginsenoside conversion and antioxidant effects. J. Ginseng Res..

[28] Banothu V., Neelagiri C., Adepally U., Lingam J., Bommareddy K. (2017). Phytochemical screening and evaluation of in vitro antioxidant and antimicrobial activities of the indigenous medicinal plant Albizia odoratissima. Pharm. Biol..

[29] Kwun S.Y., Bae Y.W., Yoon J.A., Park E.H., Kim M.D. (2020). Isolation of acid tolerant lactic acid bacteria and evaluation of α-glucosidase inhibitory activity. Food Sci. Biotechnol..

[30] Chen T., Wang L., Li Q., Long Y., Lin Y., Yin J., Zeng Y., Huang L., Yao T., Abbasi M.N. (2020). Functional probiotics of lactic acid bacteria from Hu sheep milk. BMC Microbiol..

[31] Fadare O.S., Singh V., Enabulele O.I., Shittu O.H., Pradhan D. (2021). In vitro evaluation of the synbiotic effect of probiotic *Lactobacillus* strains and garlic extract against Salmonella species. LWT.

[32] Rajab S., Tabandeh F., Shahraky M.K., Alahyaribeik S. (2020). The effect of *Lactobacillus* cell size on its probiotic characteristics. Anaerobe.

[33] Kebouchi M., Galia W., Genay M., Soligot C., LeComte X., Awussi A.A., Perrin C., Roux E., Dary-Mourot A., Le Roux Y. (2016). Implication of sortase-dependent proteins of Streptococcus thermophilus in adhesion to human intestinal epithelial cell lines and bile salt tolerance. Appl. Microbiol. Biotechnol..

[34] Cai T., Wu H., Qin J., Qiao J., Yang Y., Wu Y., Qiao D., Xu H., Cao Y. (2019). In vitro evaluation by PCA and AHP of potential antidiabetic properties of lactic acid bacteria isolated from traditional fermented food. LWT.

[35] Chen P., Zhang Q., Dang H., Liu X., Tian F., Zhao J., Chen Y., Zhang H., Chen W. (2014). Screening for potential new probiotic based on probiotic properties and α-glucosidase inhibitory activity. Food Control.

[36] Han Q., Kong B., Chen Q., Sun F., Zhang H. (2017). In vitro comparison of probiotic properties of lactic acid bacteria isolated from Harbin dry sausages and selected probiotics. J. Funct. Foods.

[37] Jara J., Pérez-Ramos A., del Solar G., Rodríguez J.M., Fernández L., Orgaz B. (2020). Role of *Lactobacillus* biofilms in Listeria monocytogenes adhesion to glass surfaces. Int. J. Food Microbiol..

[38] Ma J., Wang W., Sun C., Gu L., Liu Z., Yu W., Chen L., Jiang Z., Hou J. (2020). Effects of environmental stresses on the physiological characteristics, adhesion ability and pathogen adhesion inhibition of *Lactobacillus plantarum* KLDS 1.0328. Process Biochem..

[39] Xu Y., Zhou T., Tang H., Li X., Chen Y., Zhang L., Zhang J. (2019). Probiotic potential and amylolytic properties of lactic acid bacteria isolated from Chinese fermented cereal foods. Food Control.

[40] Zhao G., Cui M., Wang M., Chen W., Li J., Yao Y. (2020). The correlation between colonization and the biological properties of Lactobacillus sp.. Food Biosci..

[41] Abushelaibi A., Al-Mahadin S., El-Tarabily K., Shah N.P., Ayyash M. (2017). Characterization of potential probiotic lactic acid bacteria isolated from camel milk. LWT.

[42] Rocha-Mendoza D., Kosmerl E., Miyagusuku-Cruzado G., Giusti M.M., Jiménez-Flores R., García-Cano I. (2020). Growth of lactic acid bacteria in milk phospholipids enhances their adhesion to Caco-2 cells. J. Dairy Sci..

[43] Kalita D., Holm D.G., LaBarbera D.V., Petrash J.M., Jayanty S.S. (2018). Inhibition of α-glucosidase, α-amylase, and aldose reductase by potato polyphenolic compounds. PLoS ONE.

[44] Zhong H., Abdullah, Zhang Y., Zhao M., Zhang J., Zhang H., Xi Y., Cai H., Feng F. (2020). Screening of novel potential antidiabetic *Lactobacillus plantarum* strains based on in vitro and in vivo investigations. LWT.

[45] Rivero-Pino F., Espejo-Carpio F.J., Guadix E.M. (2020). Production and identification of dipeptidyl peptidase IV (DPP-IV) inhibitory peptides from discarded Sardine pilchardus protein. Food Chem..

[46] Kaprasob R., Sarkar D., Kerdchoechuen O., Laohakunjit N., Khanongnuch C., Shetty K. (2019). Beneficial lactic acid bacteria based bioprocessing of cashew apple juice for targeting antioxidant nutraceutical inhibitors as relevant antidotes to type 2 diabetes. Process Biochem..

[47] Tang W., Xing Z., Li C., Wang J., Wang Y. (2017). Molecular mechanisms and in vitro antioxidant effects of *Lactobacillus plantarum* MA2. Food Chem..

[48] Martindale J.L., Holbrook N.J. (2002). Cellular response to oxidative stress: Signaling for suicide and survival. J. Cell. Physiol..

[49] Jia J., Zhang X., Hu Y.-S., Wu Y., Wang Q.-Z., Li N.-N., Guo Q.-C., Dong X.-C. (2009). Evaluation of in vivo antioxidant activities of Ganoderma lucidum polysaccharides in STZ-diabetic rats. Food Chem..

[50] Meng L., Zhu X., Tuo Y., Zhang H., Li Y., Xu C., Mu G., Jiang S. (2021). Reducing antigenicity of β-lactoglobulin, probiotic properties and safety evaluation of *Lactobacillus plantarum* AHQ-14 and *Lactobacillus bulgaricus* BD0390. Food Biosci..

